# Bottles to trees: Plastic beverage bottles as an alternative nursery growing container for reforestation in developing countries

**DOI:** 10.1371/journal.pone.0177904

**Published:** 2017-05-31

**Authors:** Safiullah Khurram, Owen T. Burney, Robert C. Morrissey, Douglass F. Jacobs

**Affiliations:** 1 Hardwood Tree Improvement and Regeneration Center, Department of Forestry and Natural Resources, Purdue University, West Lafayette, Indiana, United States of America; 2 John T. Harrington Forestry Research Center. New Mexico State University. Mora, New Mexico, United States of America; Youngstown State University, UNITED STATES

## Abstract

Reforestation is needed globally to help restore degraded sites, combat desertification, protect watersheds, and provide forest products. This involves planting forest tree seedlings grown in local nurseries, but technologies to produce quality seedlings are lacking in developing countries. Modern nursery containers used to propagate seedlings have internal-surface barriers (ribs or ridges) or side-slits to prevent root spiraling. These are cost prohibitive or unavailable in developing countries and so polybags (plastic bags) are more commonly used, despite their tendency to produce seedlings with deformed root systems that have less potential to establish on field sites. Discarded plastic bottles, which are readily available worldwide, may be a feasible alternative for seedling propagation. We conducted two experiments to assess the potential of repurposed plastic beverage bottles to grow quality trees: 1) *Container Comparison*–to evaluate Arizona walnut (*Juglans major* [Toor.] Heller) and Afghan pine (*Pinus eldarica* Medw.) seedling root and shoot development in two plastic bottle types compared to modern nursery containers and polybags, and 2) *Bottle Modification*–to examine the effects of root spiraling prevention techniques (side-slits, internal-ridges, and control) and container opacity (green, black, and clear) on Afghan pine seedling morphological attributes. Nursery growth and first-year seedling field performance were evaluated for both experiments. In experiment one, seedlings of both species had fewer spiraled roots in bottle containers compared to polybags. Arizona walnut had more fibrous root systems in polybags, while Afghan pine root system fibrosity was greatest in bottle containers. First-year field performance of both species was not affected by container type. In experiment two, less spiraled roots occurred in containers with air-slits and interior-ridges compared to the control. The effects of container opacity on seedling morphology were inconsistent. Root spiral prevention and opacity had no influence on Afghan pine one-year survival, field height and diameter, with the exception of opacity for height growth, whereby seedlings grown in green containers were taller than those grown in black containers, but seedlings grown in clear containers were similar to both. Our results provide the first evidence that plastic bottle containers may provide an effective alternative for production of high quality seedlings, which may benefit agroforestry, reforestation, restoration, and conservation programs in developing countries.

## Introduction

Deforestation is an increasingly global issue, particularly in developing countries. Resource limitations are major drivers of deforestation because many people subsist by exploiting forest resources for agricultural expansion, livestock, and fuel [1]. Effective systems often do not exist to adequately reforest after disturbance, and governments of these countries are generally unable to provide adequate services or effectively implement policies designed to promote the sustainability and conservation of natural resources [1]. Thus, many reforestation programs in developing countries are unsuccessful due to limited resources and expertise, and a lack of quality planting materials [2–5].

Despite these challenges, ambition is high worldwide for promotion of reforestation programs that lead to successful site restoration and rehabilitation [6]. Quality nursery seedlings suitable for the environmental conditions of the outplanting site are vital to success of these programs. Extensive research suggests that nursery operations play a significant role in seedling quality and outplanting performance [7–10]. The Target Plant Concept defines the physiological (e.g., cold hardiness and root growth potential) and morphological (e.g., shoot to root ratio and stem diameter) characteristics of seedlings grown in a nursery that promote outplanting performance [11].

However, the Target Plant Concept or ‘fitness of purpose’, as it is generally defined, is not commonly considered in many forest restoration programs in developing regions because of limited resources [12]. For example, production nurseries often establish bare-root crops or use polybags (plastic bags) because they cannot afford appropriate container types [5,13]. Polybags filled with native topsoil and kept on bare-ground is a common practice in nurseries in many developing countries [5,14]. Although polybags are readily available and cheap, seedlings grown in polybags with smooth inner-surfaces and heavy topsoil are prone to root deformities that often result in poor outplanting performance [4, 14–16], especially compared to modern nursery containers that have internal surface barriers (ribs or ridges) or side-slits to prevent root spiraling [17]. Root deformities, such as spiraled or J-shaped root systems, may reduce survival, stress resistance, water and nutrient uptake, vigor, and mechanical stability after outplanting [18–24]. Cedamon et al. [25] reported highly deformed root systems of two species grown in polybags compared to those grown in a standard container type (hiko trays). Root egress from bags and growth into the soil below the polybag is another issue that may cause uneven seedling growth and root system damage during lifting [26]. Thus, use of polybags will likely result in lower quality seedlings with less potential to establish and perform in harsh outplanting sites [4, 14–16]. Polybags are also not reusable [13], creating a negative impact on the environment [27–28].

Many developing countries are concurrently facing severe environmental problems that can be ameliorated by planting trees in restoration projects [29]. Reforestation and restoration success in developing countries could be vastly improved with a readily available and cheap alternative to modern nursery containers. International bottling companies produce bottles for water and soft drinks; these bottles are generally only used for beverage consumption by a single user, after which the bottle is discarded. In 2009, approximately 120 billion plastic water bottles (excluding carbonated beverages such as sodas) were used worldwide [30]. Despite concentrated efforts, plastic bottles are often considered waste where recycling and waste management are limited and are discarded in streets, waterways, and open areas. Plastic water bottles may provide an inexpensive and re-usable alternative for growing containers with the two-fold advantage of reducing waste and extending the life of these products. The texture, pattern, color, and thickness of these plastic bottles vary greatly, however, and so before they can be used operationally, research is needed to examine seedling growth and development in these bottles used as nursery containers.

The objective of this study was to develop and evaluate technology that allows for the repurpose of plastic beverage bottles for use as nursery containers for growing quality native trees for agroforestry, reforestation, restoration, and conservation programs. We assessed seedling growth and morphology in two separate experiments: 1) *Container Comparison–*to evaluate seedling root and shoot development in two plastic bottle types, modern nursery containers, and polybags; and 2) *Bottle Modification*–to examine the effects of root spiraling control techniques and container opacity on seedling morphology. For each experiment, we assessed plant quality at the end of nursery growth and seedling survival and performance in the field for one growing season.

## Material and methods

### Plant material and experimental treatments

**Fig 1:**
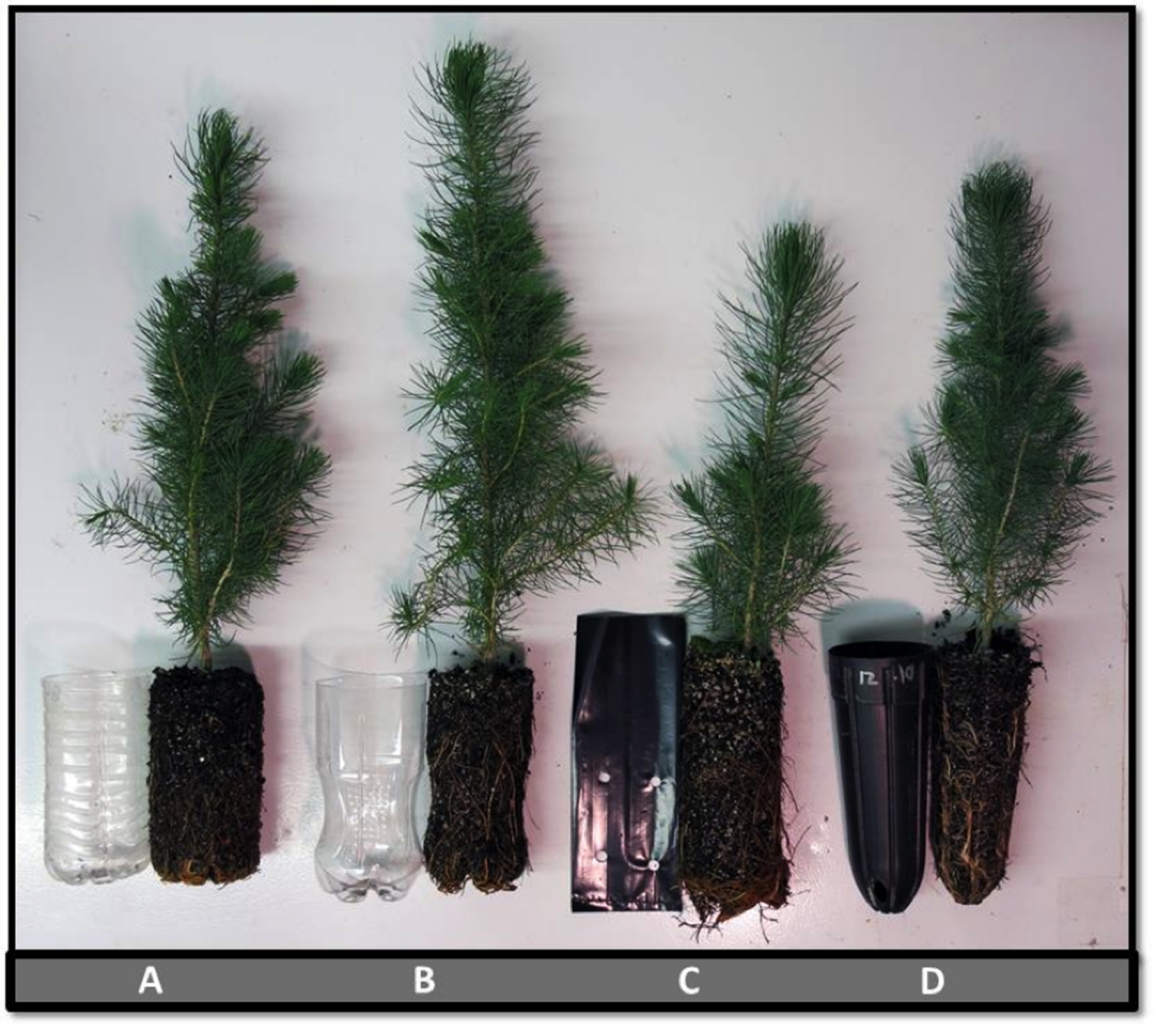
Comparison of different container types. **A) Sam’s Club® water bottle; B) Coca-Cola® bottle; C) polybag; D) modern container—Deepot™D27 –**photo by O.Burney.

**Fig 2:**
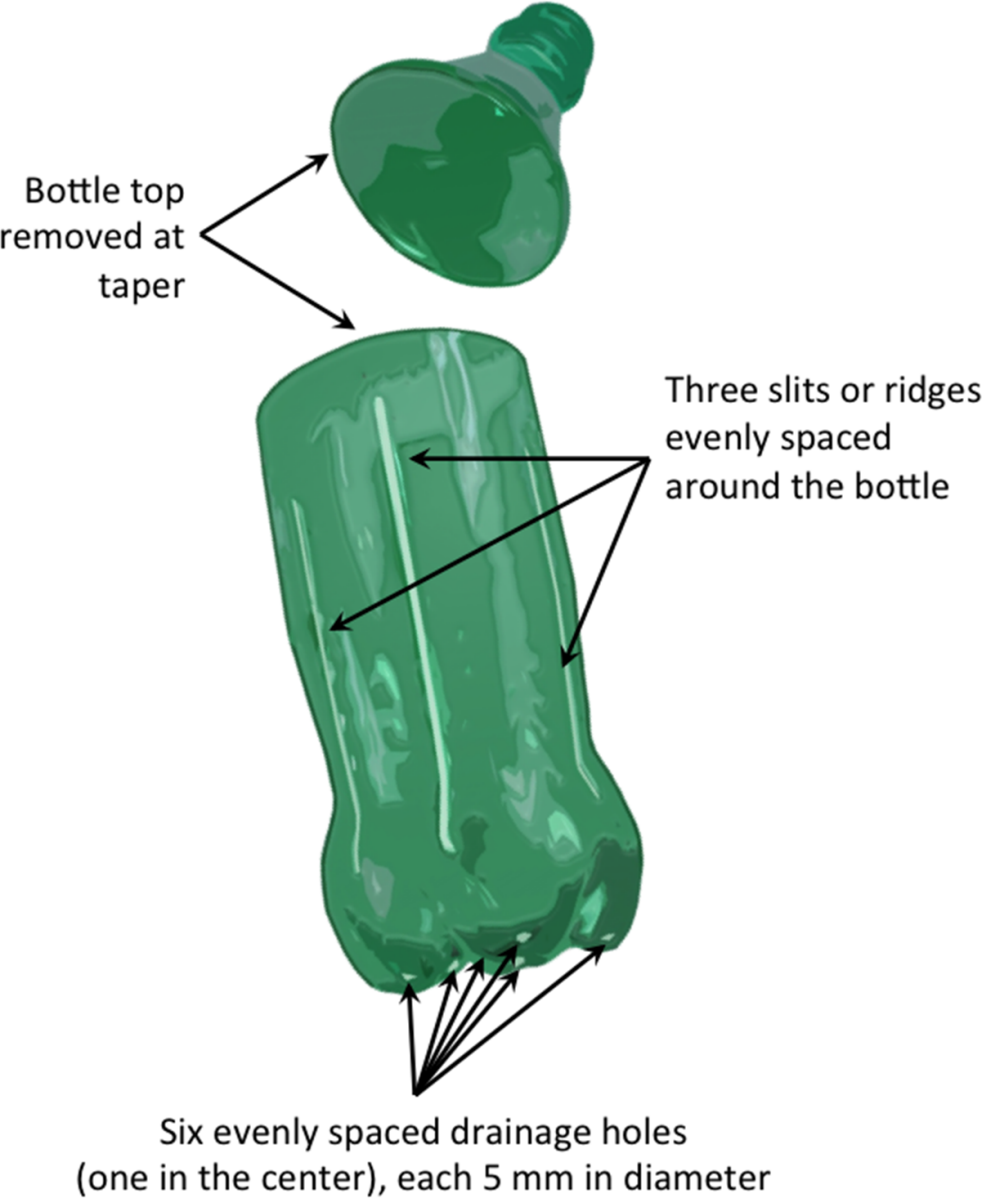
Example of plastic bottle modifications using side slits as root spiraling control method–graphic by R.Heyduck.

For the *Container Comparison* experiment, two species, Afghan pine (*Pinus eldarica* Medw.) and Arizona walnut (*Juglans major* [Toor.] Heller), were examined as separate studies. We used four container types, including Coca-Cola^®^ beverage bottles (Coke), modern Deepot^TM^ D27 containers (D27), Polyethylene polybags (polybag), and Sam’s Club^®^ water bottles (Sams) (Fig 1). The D27 containers were chosen to represent a standard industrial container type to compare with bottles and polybags. No alterations were made to the D27 containers; they had internal ribs for root spiraling prevention and holes at the base for drainage. The depth and top diameter of D27 containers were 17.8 cm and 6.4 cm, respectively. Plastic bottle containers were 0.5-L bottles from Coca-Cola^®^ and Sam’s Club^®^ bottling companies. The depth and top diameter of both types of bottle containers were 13 cm and 7 cm, respectively. Tops were removed from each bottle and six evenly-spaced holes were drilled in the bottom for drainage. Root spiraling was anticipated without using any control methods in the two bottle containers; thus, three 12-cm long vertical slits were inserted evenly around the interior perimeter of the bottle to prevent root spiraling (Fig 2). The volume of the altered bottles, D27 containers, and polybags was similar, approximately 500 cm^3^. The primary difference between the polybags and other container types was the lack of any root spiraling prevention mechanism in the polybags. The *Container Comparison* experiment was established as a randomized complete block design with four container treatments and five blocks. Each block (replicate) contained 16 seedlings per treatment combination (64 seedlings per species per block, for a total of 640 seedlings).

**Fig 3:**
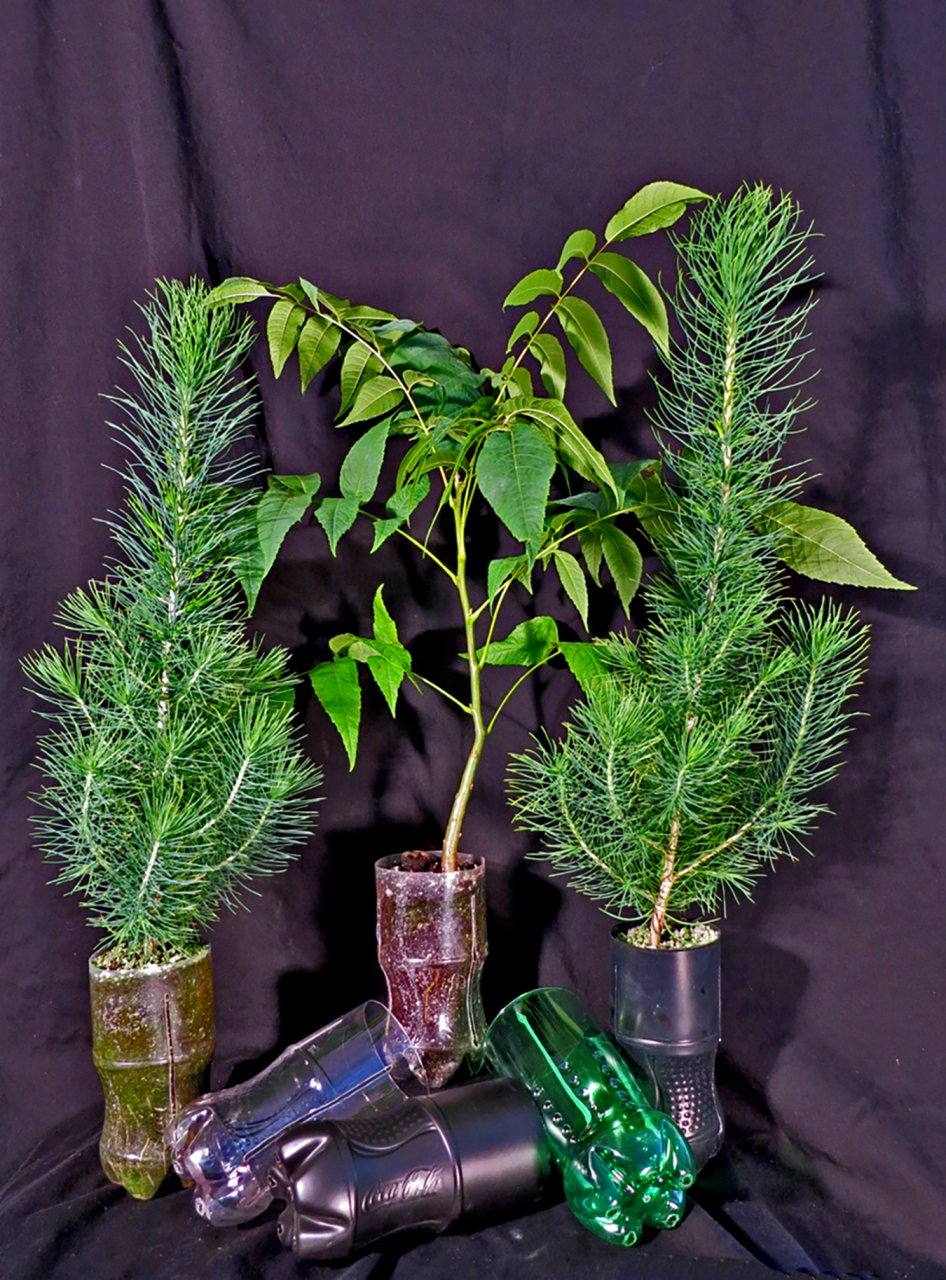
Plastic bottles used as alternative containers for seedling production–photo by R.Heyduck.

For the *Bottle Modification* experiment, we used only Afghan pine. We examined the effects of three levels of bottle opacity (clear, green, and opaque) and three methods of root spiraling prevention (side-slits, internal ridges, and control with no alterations) (Fig 3). The opaque bottles were typical Coca-Cola^®^ bottles coated with black paint, while the green and clear bottles were the original bottles of Sprite and Coca-Cola^®^ beverages, respectively. All three colors were considered as opacity treatments to test the overall seedling performance, differences in soil media temperature, and algae development. In addition to the unaltered bottles used in the control treatment, we used a side-slit treatment, three 12-cm long vertical side-slits evenly spaced around the circumference of the bottle, and an internal ridge treatment, which entailed the use of three 12-cm long internal ridges evenly spaced around the interior perimeter of the bottle using silicon adhesive (Fig 2). This experiment was completely randomized with a 3 × 3 factorial structure (bottle opacity × root spiraling prevention); the nine treatment combinations each had 60 seedlings (540 seedlings total). Rather than organizing the containers side-by-side as we did in the *Bottle Comparison* experiment, bottles for the *Bottle Modification* experiment were equally spaced (10 cm spaces) to ensure more uniform lighting and air movement around individual bottles.

For both experiments and species, seeds were sown in the first week of April 2013 at the John T. Harrington Forestry Research Center in Mora, New Mexico, USA (35° 58’ N, 105° 20’ W; 2207 m ASL). Seedlings were cultivated for one growing season (2013) in a traditional greenhouse nursery with heating and cooling systems. Daytime and nighttime temperatures were maintained at 21–26°C and 18–22°C, respectively. Relative humidity ranged between 60 to 80% during the growing phase. Artificial lighting was used to supplement natural light to ensure a minimum12-hour photoperiod. The growing medium was a mixture of peat, vermiculite, and perlite at a ratio of 2:1:1, respectively. Containers were kept moist until seed germination, after which irrigation was based on a gravimetric method [31–32]. Gravimetric weights, measured on a daily basis, were taken from subsamples from each treatment to determine the irrigation schedule. Treatments were irrigated when total weights (sum of container, dry media, and water at field capacity) fell below 80% as defined by Dumroese et al. [32]. This equates to an approximate reduction of 64% of the total water weight of the container before irrigation commenced. Irrigation water was maintained at a pH of 6.0 to 6.5 by mixing hydro phosphoric acid (H_3_PO_4_) as necessary.

Water-soluble fertilizer was applied in all treatments at three different growing stages (i.e., germination, rapid growth, and hardening) based on current operational nursery programs at New Mexico State University and University of Idaho. Fertilizer was applied in every other irrigation for 30–45 minutes and the application rate ranged from 25 to 150 mg/L. Fertilization started in the beginning of June after seed germination, and it ceased in early November in preparation for hardening off and storage. In early June, after seed germination, fertilizer (NPK10:30:20) was applied at a rate of 25 mg/L. To promote rapid shoot growth after germination (mid-June), fertilizer (NPK 21:5:20) was applied at an increasing rate until a peak rate of 125 mg/L was achieved in late August, after which it progressively declined to 25 mg/L. To slow growth and prepare the seedlings for hardening off and storage, fertilizer (NPK 4:25:35) was applied for five weeks from late-September to early-November. To initiate the hardening process in September, greenhouse lighting and temperatures were reduced using shade cloth, greenhouse vents, and cooling pads. At the end of the first growing season (December 2013), seedlings were stored in a walk-in cooler (1°C) to maintain dormancy and prevent winter cold injury. The gravimetric weight method was used to assess dry down of stored seedlings; no irrigation was required based on a recommended target of 65% field capacity.

Seedlings for both experiments were outplanted at the John T. Harrington Forestry Research Center in June 2014. The site was free of vegetation with well-drained Brycan loam soils [33] and was mechanically prepared using disking (20 cm depth) with a tractor before planting; it had been managed for an Alfalfa experiment trial about five years prior to planting. There was no precipitation one month prior or seven weeks after outplanting. To ensure at least modest survival under these exceptionally dry outplanting conditions, a field sprinkler irrigation system was initially used to water seedlings twice a week for a two-hour period for seven weeks. Competing vegetation was mechanically removed during the early growing season. Outplanting of the *Container Comparison* experiment seedlings followed the same experimental design as the nursery component (randomized complete block design), but the *Bottle Modification* experiment was modified from a completely randomized design to a randomized complete block design with a 3 × 3 factorial structure (spiral prevention × opacity) replicated with three blocks. This modification to a blocking structure was necessary to account for the potential environmental differences at the outplanting site.

### Measurements

Seedling measurements were the same for both experiments. Seedling height and root collar diameter (RCD) were recorded when seedlings were destructively sampled to evaluate seedling morphology. We harvested four seedlings per treatment combination per block for the *Container Comparison* experiment and fifteen seedlings per treatment combination for the *Bottle Modification* experiment for measurements in August and November.

Destructively sampled seedlings were evaluated for shoot and root morphology and architecture. Destructive measurements included shoot and root volumes, shoot and root dry mass, and the number of lateral roots, spiraled roots, and controlled roots. Spiraled roots were defined as roots that began to grow nearly parallel to the ground without interruption after contact with the container wall for two-thirds or more of the container circumference. Controlled roots were those that began to spiral, but then changed direction downward towards the bottom of the container. Additionally, the fresh mass of algae growth on the inner container walls of the emptied containers from the *Bottle Modification* experiment was scrapped off and weighed for the first destructive sampling period (August); algae growth on the bottles will make them more difficult to clean for re-use and may even limit re-use of the bottles.

Seedlings were lifted from containers and roots were carefully washed to remove the growing medium. Root and shoot volumes were measured using the water displacement method [19]. Shoots were separated from the root system at the root collar and placed into individual paper bags to determine their dry weight. The lateral and tap roots for each segment were placed into paper bags for drying. All plant material, shoots and separated roots, was placed into a drying oven at 70°C for 48 hours and then weighed to the nearest 0.10 g.

Root architecture of individual seedlings was assessed systematically. Total root length was measured from the root collar to the end of the taproot; if multiple taproots existed, the longest was used for measurements. The number of first order lateral roots, spiraled lateral roots, and controlled roots were counted.

Root fibrosity, root to shoot ratio, and seedling sturdiness quotient were used to evaluate the effect of container type and container modifications on seedling growth and quality. Root fibrosity was calculated as the percent of root dry mass represented by the lateral roots [34]:

$$Root\;fibrosity=\left(\frac{Lateral\;roots\;dry\;weight\;(g)}{Total\;root\;dry\;weight\;(g)}\right)\times \;Number\;of\;lateral\;roots$$

For root to shoot ratio, root dry mass was divided by shoot dry mass, and the sturdiness quotient of seedlings was calculated as shoot height (cm) divided by root collar diameter (mm) [35].

To compare the effect of treatment combinations on substrate temperature for the *Bottle Modification* experiment, container substrate temperature was measured using Thermochron® iButton Data Loggers (Maxim Integrated ™, San Jose, California, USA; [36]. We used a complete randomized design with a 2×3 factorial structure (slits, no-slits × black, clear, green) with four replications per treatment combination (n = 24). Temperature estimates reflect the daily maximum temperature from hourly measurements each day over a 13-day period.

In the outplanting phase, seedlings were measured for height and ground line diameter at the time of planting (June 2014) and at the end of the growing season (November 2014) for both experiments. Survival was 100% for all seedlings at the end of one growing season after outplanting. Relative height and diameter growth were based on the change in absolute height or diameter between specific time periods relative to the initial height or diameter of the seedling at the time of outplanting.

### Statistical analysis

All data were analyzed using the mixed model procedure (PROC MIXED) in SAS (SAS Institute Inc., Cary, NC, USA) with *α* = 0.05. For the *Container Comparison* experiment, effects of container types on seedling morphology were analyzed using analysis of variance (ANOVA) for a single factor randomized complete block design for each species, separately, for both the greenhouse and field components. When significant effects were detected within main effects, Fisher’s Least Significant Difference (LSD) test was performed to detect significant differences between means at *P* <0.05. Residuals of all response variables were checked for normality and constant variance to ensure they met ANOVA assumptions. Similarly, for the *Bottle Modification* experiment, ANOVA for a two-factor randomized complete block design was used to examine the effects of root spiraling prevention methods and opacity treatment on seedling morphological parameters.

## Results

### Container comparison

Container type influenced morphological responses for Arizona walnut seedlings in the nursery phase (Table 1). At the first sampling period in August, shoot height, shoot dry biomass, and root volume were greater in D27 containers compared to Coke bottles and polybags, while Sams bottles did not differ among treatments. Coke and Sams bottles produced seedlings with lower FOLR dry mass than D27 containers, while polybags had greater FOLR dry mass compared to Coke bottles. Arizona walnut seedlings produced more fibrous root systems in polybags and D27 compared to Coke bottles, while seedlings grown in Sams bottles were not different from all other containers. Total dry mass of seedlings grown in D27 containers was greater compared to all other containers.

**Table 1:** Mean estimates of shoot, root collar diameter (RCD), first-order lateral roots (FOLR), and overall root, and total tree morphological parameters for Arizona walnut seedlings in August and November sampling periods.

		August												November											
		D27			Coke			Sams			Polybag			D27			Coke			Sams			Polybag		
Shoot																									
	Shoot height (cm)	34.2	(3.2)	a	28.2	(1.0)	b	28.9	(1.7)	ab	27.8	(2.2)	b	36.0	(1.4)	a	28.9	(3.3)	b	29.9	(1.0)	b	31.0	(2.2)	ab
	Shoot volume (cm^3^)	34.1	(0.9)	a	29.1	(1.4)	a	32.0	(2.9)	a	29.4	(2.7)	a	12.0	(0.9)	a	10.4	(1.8)	a	9.4	(1.1)	a	10.4	(1.3)	a
	Shoot dry mass (g)	7.8	(0.7)	a	5.7	(0.4)	b	6.6	(0.6)	ab	5.7	(0.7)	b	6.6	(0.5)	a	6.4	(1.0)	a	5.2	(0.4)	a	6.1	(0.8)	a
Root																									
	RCD (mm)	6.9	(0.4)	a	6.2	(0.2)	a	6.5	(0.4)	a	6.2	(0.1)	a	12.3	(0.6)	a	12.9	(0.9)	a	11.5	(0.3)	a	11.7	(0.7)	a
	Root volume (cm^3^)	24.2	(1.8)	a	18.1	(1.7)	b	20.5	(1.7)	ab	19.9	(2.2)	b	72.3	(4.6)	a	63.0	(6.4)	ab	57.0	(1.7)	b	73.2	(8.6)	a
	Root dry mass (g)	5.9	(0.5)	a	4.1	(0.6)	b	4.5	(0.5)	b	4.0	(0.6)	b	32.3	(2.7)	a	29.6	(2.6)	a	24.4	(1.9)	a	32.5	(4.9)	a
	Root fibrosity	6.4	(0.4)	a	3.3	(0.5)	b	4.6	(0.7)	ab	6.5	(0.6)	a	3.5	(0.7)	ab	2.3	(0.3)	b	3.0	(0.4)	ab	4.1	(0.5)	a
	FOLR dry mass (g)	0.8	(0.1)	a	0.3	(0.0)	c	0.4	(0.1)	bc	0.6	(0.1)	ab	4.8	(1.2)	a	3.1	(0.6)	a	3.4	(0.7)	a	4.7	(0.8)	a
	Total FOLRs (#)	44.9	(2.0)	a	37.7	(1.6)	a	43.9	(5.0)	a	44.1	(5.1)	a	25.7	(1.9)	b	26.8	(1.6)	ab	24.4	(2.1)	b	32.1	(3.4)	a
	Spiral roots (#)	0.2	(0.1)	a	0.8	(0.1)	a	0.9	(0.2)	a	1.9	(1.1)	a	1.9	(0.9)	ab	0.8	(0.4)	b	0.9	(0.2)	b	3.8	(0.6)	a
	Controlled roots (#)	1.5	(0.4)	a	0.4	(0.2)	b	0.7	(0.1)	ab	0.0	NA	b	3.0	(0.5)	a	2.2	(0.7)	ab	1.2	(0.4)	bc	0.4	(0.2)	c
Tree																									
	Total tree dry mass (g)	13.3	(1.1)	a	9.4	(1.0)	b	10.7	(1.0)	b	9.3	(1.3)	b	39.3	(3.0)	a	36.4	(3.5)	a	30.0	(1.9)	a	39.0	(5.6)	a
	Sturdiness quotient	4.9	(0.3)	a	4.5	(0.1)	ab	4.3	(0.1)	b	4.4	(0.3)	ab	3.0	(0.2)	a	2.2	(0.2)	b	2.7	(0.1)	a	2.7	(0.1)	a
	Root:Shoot	0.7	(0.0)	a	0.8	(0.1)	a	0.7	(0.1)	a	0.7	(0.0)	a	5.2	(0.4)	a	5.6	(0.9)	a	5.3	(0.6)	a	5.8	(0.3)	a

Container types: Deepot^TM^ D27 (D27), Coca-Cola^®^ bottle (Coke), Sam’s Club^®^ bottle (Sams), and Polyethylene polybag (Polybag). Container types with different lower-case letters within rows of each measurement period are significantly different (α = 0.05) according to Fisher’s LSD test. Standard errors are in parentheses.

By November, only shoot height differed for shoot morphological responses among Arizona walnut container treatments (Table 1). Seedlings grown in the D27 containers had greater heights compared to those grown in Coke and Sams bottles, although they did not differ from seedlings grown in polybags (Table 1). Root morphological responses continued to show instances of differences among treatments. The number of spiraled roots of seedlings grown in polybags was greater compared to Coke and Sams bottles, although seedlings grown in D27 containers were not different than the other three treatments. However, the number of spiraled roots in the D27, Coke, and Sams containers was almost half of that found in the polybags. The number of controlled roots was highest for seedlings grown in D27 and Coke containers, and those grown in Sams bottles were not different than Coke bottles or polybags.

Afghan pine shoot and root responses also exhibited differences among container types in both sampling periods (Table 2). Shoot height was greater at the initial sampling period for seedlings grown in the Sams bottles compared to polybags, while seedlings grown in Coke bottles and D27 containers did not differ among treatments. Root fibrosity was lower for seedlings grown in D27 containers compared to all other treatments with the exception of the polybag treatment. The number of FOLRs was higher for seedlings grown in Coke bottles compared to D27 and polybag containers, although seedlings grown in Coke and Sams bottles did not differ. Total tree measures showed that sturdiness quotient was greater in seedlings grown in Sams bottles compared to those grown in D27 containers, but they did not differ from seedlings of the Coke or polybag treatments.

**Table 2:** Mean estimates of shoot, root collar diameter (RCD), first-order lateral roots (FOLR), and overall root, and total tree morphological parameters for Afghan pine seedlings in August and November sampling periods.

		August												November											
		D27			Coke			Sams			Polybag			D27			Coke			Sams			Polybag		
Shoot																									
	Shoot height (cm)	15.2	(1.0)	ab	15.7	(0.5)	ab	16.4	(0.9)	a	15.0	(0.3)	b	32.4	(2.6)	b	36.1	(2.3)	ab	37.2	(1.7)	a	34.5	(2.5)	ab
	Shoot volume (cm^3^)	5.5	(0.8)	ab	6.2	(0.4)	ab	6.3	(0.5)	a	5.0	(0.4)	b	31.7	(4.4)	b	38.9	(5.3)	ab	42.0	(3.0)	a	33.2	(2.9)	b
	Shoot dry mass (g)	1.0	(0.1)	a	1.1	(0.1)	a	1.1	(0.1)	a	0.9	(0.0)	a	6.7	(1.2)	b	8.2	(1.1)	ab	8.9	(0.4)	a	7.0	(0.5)	b
Root																									
	RCD (mm)	2.3	(0.1)	a	2.4	(0.1)	A	2.3	(0.1)	a	2.2	(0.1)	a	5.1	(0.4)	b	5.9	(0.4)	a	6.0	(0.1)	a	5.6	(0.2)	ab
	Root volume (cm^3^)	5.1	(0.5)	a	5.0	(0.2)	a	5.3	(0.3)	a	4.7	(0.4)	a	24.2	(4.0)	a	30.5	(4.1)	a	29.0	(1.6)	a	27.1	(3.3)	a
	Root dry mass (g)	0.4	(0.0)	a	0.3	(0.0)	a	0.4	(0.0)	a	0.4	(0.0)	a	2.9	(0.6)	a	3.6	(0.6)	a	3.5	(0.2)	a	3.1	(0.2)	a
	Root fibrosity	30.1	(2.5)	b	41.0	(1.2)	a	39.3	(2.3)	a	31.4	(1.3)	b	28.6	(2.1)	c	42.3	(3.1)	ab	47.6	(5.4)	a	38.6	(2.4)	b
	FOLR dry mass (g)	0.2	(0.0)	a	0.2	(0.0)	a	0.3	(0.0)	a	0.2	(0.0)	a	2.2	(0.5)	a	2.7	(0.5)	a	2.6	(0.2)	a	2.3	(0.2)	a
	Total FOLRs (#)	46.0	(2.6)	c	58.8	(2.5)	a	53.1	(2.8)	ab	48.2	(2.0)	bc	35.1	(2.6)	c	54.4	(5.0)	ab	63.4	(8.5)	a	50.2	(4.5)	b
	Spiral roots (#)	0.0	NA	b	0.4	(0.1)	a	0.1	(0.1)	b	0.2	(0.1)	ab	0.0	NA	b	0.0	NA	b	0.0	NA	b	3.1	(0.7)	a
	Controlled roots (#)	0.2	(0.1)	a	0.2	(0.1)	a	0.2	(0.1)	a	0.0	NA	a	3.4	(0.7)	a	2.0	(0.2)	b	2.1	(0.4)	b	0.1	(0.1)	c
Tree																									
	Total tree dry mass (g)	1.4	(0.2)	a	1.4	(0.1)	a	1.4	(0.1)	a	1.3	(0.1)	a	9.6	(1.7)	b	11.8	(1.7)	ab	12.4	(0.6)	a	10.1	(0.5)	ab
	Sturdiness quotient	6.6	(0.1)	b	6.7	(0.2)	ab	7.3	(0.4)	a	7.0	(0.4)	ab	6.2	(0.3)	a	6.3	(0.2)	a	6.3	(0.3)	a	6.1	(0.3)	a
	Root:Shoot	0.4	(0.0)	a	0.3	(0.0)	a	0.3	(0.0)	a	0.4	(0.1)	a	0.4	(0.0)	a	0.4	(0.0)	a	0.4	(0.0)	a	0.4	(0.0)	a

Container types: Deepot^TM^ D27 (D27), Coca-Cola^®^ bottle (Coke), Sam’s Club^®^ bottle (Sams), and Polyethylene polybag (Polybag). Container types with different lower-case letters within rows of each measurement period are significantly different (α = 0.05) according to Fisher’s LSD test. Standard errors are in parentheses.

By November, Afghan pine height response was greater in Sams bottles compared to the D27 containers (Table 2); heights for Coke and polybags did not differ among treatments. Seedling shoot volume and dry mass were greater in Sams bottles compared to those grown in D27 and polybags; seedlings grown in Coke bottles did not differ among treatments. Differences in root fibrosity and number of FOLRs in August persisted through November; although seedlings grown in polybags were no longer lower, seedlings grown in D27 containers again exhibited less fibrous root systems and fewer FOLRs compared to all other treatments. Similar to Arizona walnut, the number of spiraled roots was greater for seedlings grown in polybags compared to all other container types. Total tree dry mass was the only total tree response to show differences among container types; seedlings grown in Sams bottles had greater total dry mass compared to D27.

**Fig 4:**
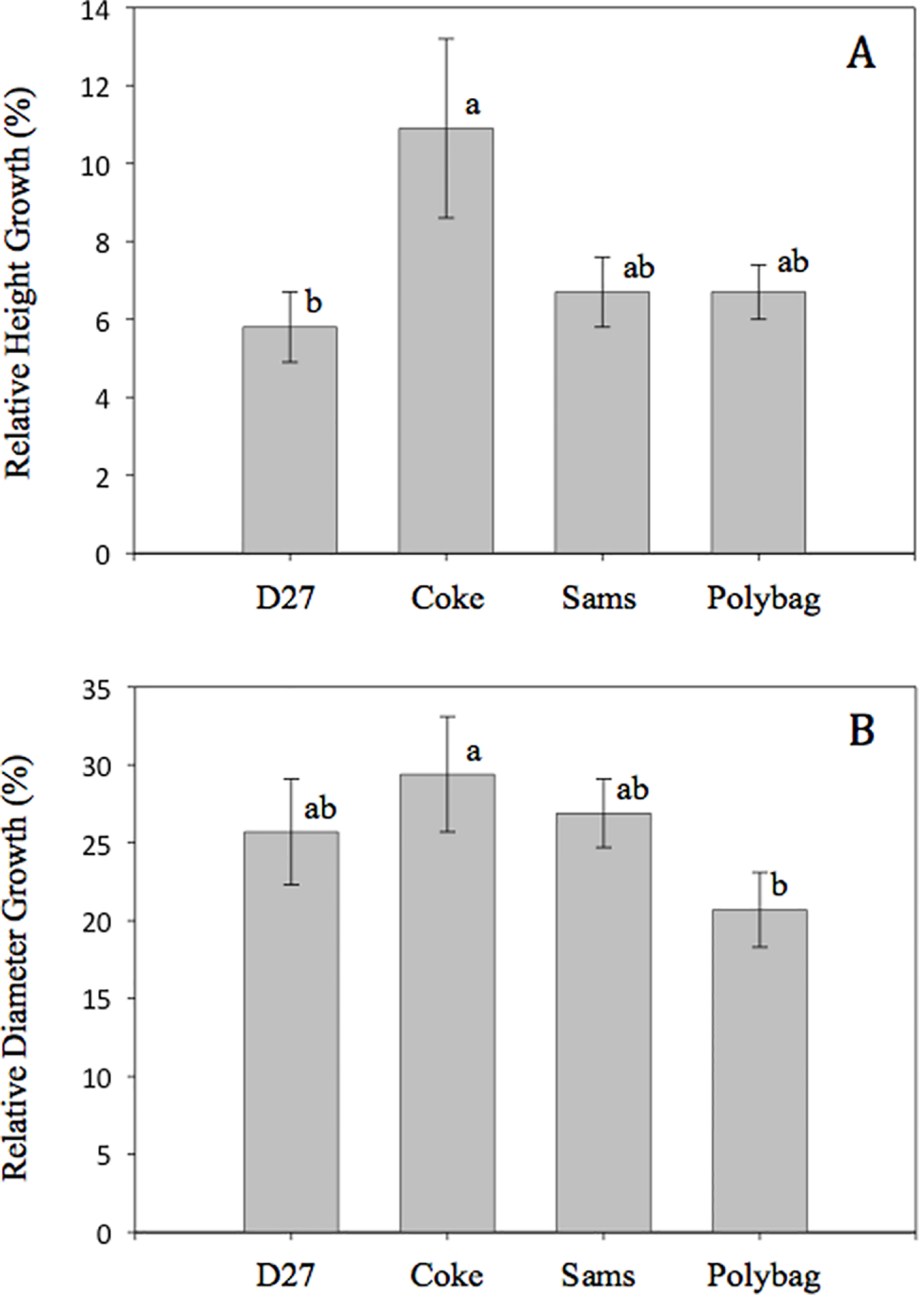
**Arizona walnut seedling field relative height (A) and root collar diameter (B) growth (Means ± SE) following the first year of outplanting.** Container types: Coca-Cola^®^ bottles (Coke), Deepot^TM^ D27 containers (D27), polyethylene polybags (Polybag), and Sam’s Club^®^ bottles (Sams). Means (±SE) not accompanied by the same lowercase letters are significantly different (α = 0.05) according to Fisher’s LSD test.

**Fig 5:**
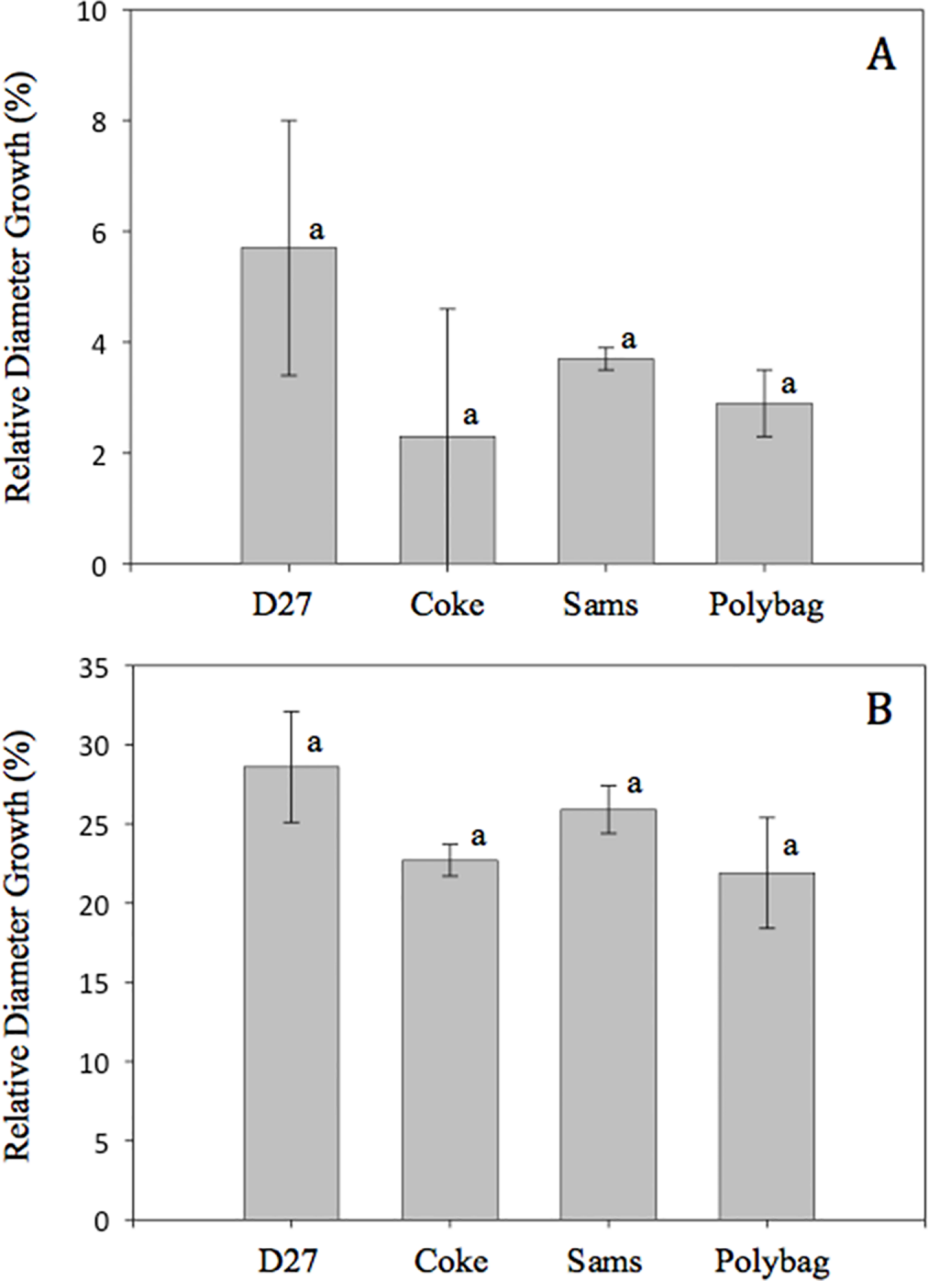
**Afghan pine seedling field relative height (A) and root collar diameter (B) growth (Means ± SE) for seedlings grown in the nursery for one growing season in four container types: Coca-Cola**^**®**^ **bottles (Coke), Deepot**^**TM**^ **D27 containers (D27), polyethylene polybags (Polybag), and Sam’s Club**^**®**^ **bottles (Sams).** Means (±SE) not accompanied by the same lowercase letters are significantly different (α = 0.05) according to Fisher’s LSD test.

At the end of the first growing season after outplanting, November 2014, Arizona walnut relative height and diameter growth was affected by container type (Fig 4; *P* = 0.006), while Afghan pine seedlings exhibited no differences by container type after outplanting (Fig 5). Arizona walnut seedlings grown in Coke bottles had greater relative height growth (11%) compared to the D27 containers (6%), and relative diameter growth of seedlings was greater in Coke bottles (29%) compared to the polybags (20%).

### Bottle modification

**Fig 6:**
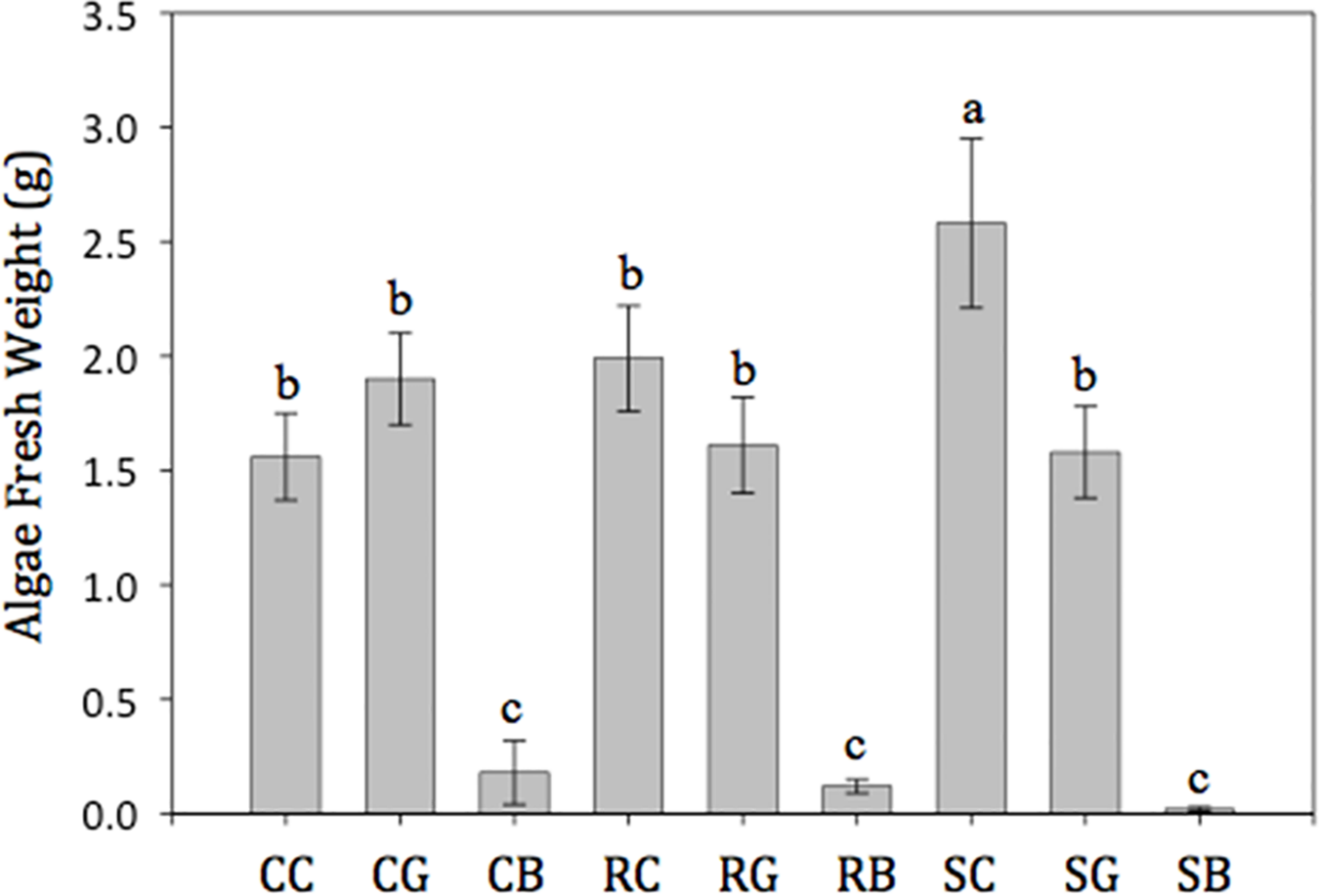
Effects of root spiraling prevention method by opacity treatment combinations on algae growth on inner container walls. The data were collected during the August 2013 destructive sampling period. The interaction between root spiraling prevention method and opacity treatments was statistically significant (*P* = 0.01). Abbreviations are: CB (control with black color), CC (control with clear color), CG (control with green color), RB (internal ridges with black color), RC (internal ridges with clear color), RG (internal ridges with green color), SB (side-slits with black color), SC (side-slits with clear color), and SG (side-slits with green color). Means (±SE) not accompanied by the same lowercase letters are significantly different (α = 0.05) according to Fisher’s LSD test.

There was only one significant interaction between root spiraling prevention techniques and opacity treatments for all morphological parameters of Afghan pine seedlings in either August or November of the nursery phase; it was for algae growth on inner container walls in August (Fig 6; *P* = 0.01). The side-slit, clear container resulted in greater algae fresh weight compared to all other containers by opacity combinations. Black containers, regardless of root spiraling prevention methods, produced lower algae fresh weight compared to clear and green containers. The interaction between container opacity and slit treatments did result in significantly different growing media temperatures (data not shown). The average daily maximum growing media temperatures were greater (*P*<0.0378) for the black bottles (36.5°C ±0.46) compared to the other two opacities by 1°C (green, 35.6°C ±0.40; clear, 35.5°C ±0.44). Additionally, bottles with side-slits were significantly (*P* <0.0005) cooler (35.1°C ±0.29) than bottles with no slits (36.6°C ±0.27).

Root spiraling prevention treatments had little impact on seedling performance at the initial sampling period in August (Table 3). Bottles with internal ridges resulted in greater root volumes compared to the side-slit treatment; however, the control treatment did not differ from the other two root spiraling prevention treatments for either metric. By November, the side-slit treatment resulted in increases in seedling RCD, shoot volume, and shoot dry mass compared to those in the other treatments (Table 3). Root spiraling was greater in the control treatment compared to the other treatments, and it had less controlled roots compared to the side-slit and ridge treatments. Total tree dry mass for seedlings grown in bottles with side slits was greater compared to all other treatments at the final measurement period.

**Table 3:** Mean estimates of root spiraling prevention treatments on morphological parameters of shoot, root collar diameter (RCD), first-order lateral roots (FOLR), and overall root, and total tree morphological parameters for Afghan pine seedling in August and November sampling periods.

		August									November								
		Control			Ridges			Slits			Control			Ridges			Slits		
Shoot																			
	Shoot height (cm)	15.1	(0.2)	a	15.5	(0.4)	a	15.2	(0.3)	a	40.1	(0.9)	a	40.5	(0.9)	a	41.0	(0.8)	a
	Shoot volume (cm^3^)	9.5	(0.4)	a	10.6	(0.5)	a	9.4	(0.4)	a	63.0	(2.3)	b	65.4	(3.0)	b	74.4	(2.2)	a
	Shoot dry mass (g)	1.5	(0.1)	ab	1.7	(0.1)	a	1.5	(0.1)	b	14.5	(0.5)	b	15	(0.6)	b	16.5	(0.5)	a
Root																			
	RCD (mm)	2.5	(0.0)	a	2.6	(0.1)	a	2.5	(0.0)	a	7.0	(0.1)	b	7.1	(0.1)	b	7.5	(0.1)	a
	Root volume (cm^3^)	6.7	(0.2)	b	7.4	(0.3)	a	6.3	(0.2)	b	40.6	(2.8)	a	39.7	(2.0)	a	41.4	(1.5)	a
	Root dry mass (g)	0.5	(0.0)	b	0.6	(0.0)	a	0.5	(0.0)	ab	5.6	(0.3)	a	5.8	(0.2)	a	5.7	(0.2)	a
	Root fibrosity	34.9	(1.1)	a	36.6	(1.1)	a	36.9	(1.3)	a	56.2	(2.9)	a	53.2	(2.9)	a	50.9	(2.8)	a
	FOLR dry mass (g)	0.3	(0.0)	a	0.4	(0.0)	a	0.3	(0.0)	a	4.3	(0.2)	a	4.4	(0.2)	a	4.4	(0.2)	a
	Total FOLRs (#)	56.4	(2.0)	a	58.7	(1.9)	a	57.1	(1.9)	a	75.0	(4.1)	a	70.6	(4.0)	a	66.7	(3.9)	a
	Spiral roots (#)	0.7	(0.2)	a	0.6	(0.1)	a	0.4	(0.2)	a	0.2	(0.1)	a	0.0	NA	b	0.0	NA	b
	Controlled roots (#)	0.1	(0.1)	a	0.1	(0.0)	a	0.1	(0.0)	a	0.0	NA	b	0.9	(0.2)	a	1.1	(0.2)	a
Tree																			
	Total tree dry mass (g)	2.0	(0.1)	ab	2.3	(0.1)	a	2.0	(0.1)	b	20.1	(0.6)	b	20.8	(0.8)	ab	22.2	(0.7)	a
	Sturdiness quotient	6.0	(0.1)	a	6.0	(0.1)	a	6.2	(0.1)	a	5.8	(0.1)	a	5.8	(0.1)	a	5.5	(0.1)	a
	Root:Shoot	0.3	(0.0)	a	0.4	(0.0)	a	0.4	(0.0)	a	0.4	(0.1)	a	0.5	(0.1)	a	0.3	(0.1)	a

Treatments with different lower-case letters within rows of each measurement period, August and November, are significantly different (α = 0.05) according to Fisher’s LSD test. Standard errors are in parentheses to right of mean estimate.

Opacity treatments had a more significant impact on seedling performance during both measurement periods. In August, the black container resulted in lower shoot height, volume, and dry mass compared to the other treatments (Table 4). By November, this trend persisted only in seedling shoot volume and dry mass between the clear and black containers. However, black containers produced seedlings with greater root dry mass in August; by November, seedlings grown in black containers had higher root fibrosity values compared to green containers, although they were not different than clear containers. Seedlings grown in black containers also had a higher number of FOLRs in both August and November compared to the other two treatments. In August, the root to shoot ratio was higher for seedlings grown in black containers, but they had a lower sturdiness quotient; by November, results showed that the clear containers had greater total dry mass compared to the black treatment.

**Table 4:** Mean estimates of container opacity treatments on morphological parameters of shoot, root collar diameter (RCD), first-order lateral roots (FOLR), and overall root, and total tree morphological parameters for Afghan pine seedling in August and November sampling periods.

		August									November								
		Clear			Green			Black			Clear			Green			Black		
Shoot																			
	Shoot height (cm)	15.9	(0.3)	a	15.7	(0.3)	a	14.2	(0.3)	b	40.9	(0.9)	a	41.4	(0.8)	a	39.3	(0.9)	a
	Shoot volume (cm^3^)	10.2	(0.4)	a	10.7	(0.4)	a	8.7	(0.4)	b	72.3	(2.8)	a	66.7	(2.2)	ab	63.8	(2.6)	b
	Shoot dry mass (g)	1.6	(0.1)	a	1.7	(0.1)	a	1.4	(0.1)	b	16.3	(0.6)	a	15.5	(0.4)	ab	14.3	(0.6)	b
Root																			
	RCD (mm)	2.6	(0.0)	a	2.6	(0.0)	a	2.5	(0.0)	a	7.3	(0.2)	a	7.2	(0.3)	a	7.0	(0.2)	a
	Root volume (cm^3^)	6.6	(0.2)	a	7.0	(0.3)	a	6.8	(0.2)	a	41.8	(1.7)	a	40.5	(2.8)	a	39.4	(1.9)	a
	Root dry mass (g)	0.5	(0.0)	b	0.5	(0.0)	b	0.6	(0.0)	a	5.9	(0.2)	a	5.5	(0.3)	a	5.6	(0.2)	a
	Root fibrosity	34.5	(1.1)	a	36.2	(0.9)	a	37.8	(1.4)	a	53.2	(3.1)	ab	47.1	(2.0)	b	60.1	(3.0)	a
	FOLR dry mass (g)	0.3	(0.0)	a	0.3	(0.0)	a	0.4	(0.0)	a	4.6	(0.2)	a	4.3	(0.2)	a	4.2	(0.2)	a
	Total FOLRs (#)	55.3	(2.0)	b	54.8	(1.2)	b	62.1	(2.2)	a	68.9	(3.8)	b	61.4	(3.0)	b	82.0	(4.4)	a
	Spiral roots (#)	0.5	(0.1)	ab	0.3	(0.1)	b	0.9	(0.2)	a	0.0	NA	a	0.2	(0.1)	a	0.0	NA	a
	Controlled roots (#)	0.1	(0.0)	a	0.1	(0.0)	a	0.1	(0.1)	a	0.5	(0.1)	b	1.0	(0.2)	a	0.6	(0.1)	ab
Tree																			
	Total tree dry mass (g)	2.1	(0.1)	a	2.2	(0.1)	a	2.0	(0.1)	a	22.2	(0.7)	a	21.1	(0.6)	ab	19.9	(0.8)	b
	Sturdiness quotient	6.2	(0.1)	a	6.2	(0.1)	a	5.8	(0.1)	b	5.7	(0.2)	a	5.8	(0.1)	a	5.6	(0.1)	a
	Root:Shoot	0.3	(0.0)	b	0.3	(0.0)	b	0.4	(0.0)	a	0.4	(0.0)	a	0.4	(0.0)	a	0.5	(0.1)	a

Treatments with different lower-case letters within rows of each measurement period, August and November, are significantly different (α = 0.05) according to Fisher’s LSD test. Standard errors are in parentheses to right of mean estimate.

**Fig 7:**
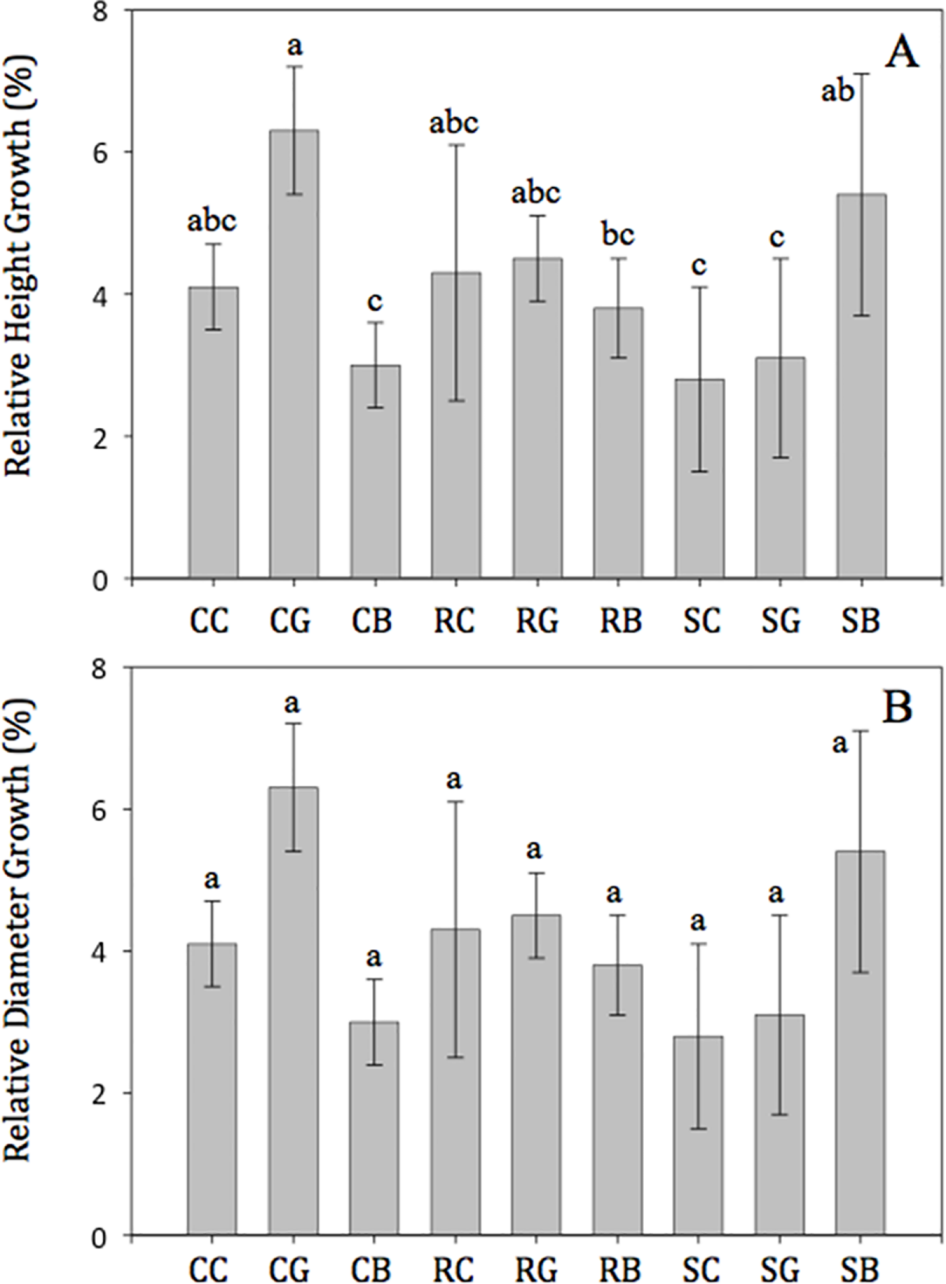
**Afghan pine seedling field relative height (A) and diameter (B) growth based on the interaction between root spiraling prevention method and opacity treatments (*P* = 0.01).** Abbreviations are: CB (control with black color), CC (control with clear color), CG (control with green color), RB (internal ridges with black color), RC (internal ridges with clear color), RG (internal ridges with green color), SB (side-slits with black color), SC (side-slits with clear color), and SG (side-slits with green color). Means (±SE) not accompanied by the same lowercase letters are significantly different (α = 0.05) according to Fisher’s LSD test. Relative growth was calculated based on the change in absolute height or diameter between final relative to the initial height or diameter of the seedling.

There were significant interactions between spiraling prevention and opacity treatments for one-year field relative height growth (Fig 7). Seedlings in the control-green treatment had the highest relative height growth (6.3%), and relative height growth was greater compared to the control-black, ridge-black, slit-clear, and slit-green treatments. All other treatment combination did not statistically differ from the control-green treatment. Outplanted seedling relative diameter growth was not affected by spiraling prevention or opacity treatments (Fig 7).

## Discussion

### Plastic bottles produce high quality seedlings

A comparison of container types showed few differences in plant growth responses at both the final nursery sampling period and one year after outplanting for either species. A key difference among container types was the increased number of spiral roots for seedlings of both species grown in polybag containers at the final nursery sampling period; however, Arizona walnut in D27 containers also exhibited abundant root spiraling. Root spiraling occurs when roots grow along the smooth, hard wall plastic of the polybag [17, 37–38], and may result in poor seedling performance, mortality, and/or toppling after outplanting [14–17]. Although no differences associated with polybags were reported after outplanting, effects from root spiraling may not be evident until several years after outplanting [17]. Unlike polybags, the other containers had either vertical slits (bottles) or ribs (D27) along the container sides, features designed to help prevent root spiraling.

Other than root spiraling, few differences were observed overall at the final nursery measurement period among treatments (root fibrosity, the number of lateral roots, and height). Afghan pine grown in plastic bottles had greater root fibrosity and number of lateral roots compared to the D27. This suggests that the side-slits may have been more effective than ribs; when lateral roots become exposed at the side-slits, their growth ceases and this stimulates the taproot to produce more lateral roots [39]. Another possible explanation is that the media temperatures in the bottles may have been more favorable for plant growth; in the *Bottle Modification* experiment, containers with air slits, regardless of color, had reduced media temperatures compared to no-slit containers.

In the outplanting phase, container type did not impact seedling shoot height and diameter growth of either species. Although the first year following planting represents the critical establishment period [40], one-year may have been insufficient to observe treatment effects. Lack of effects may have also been associated with post-planting irrigation, which was used to reduce potential mortality following the relatively late planting date.

Our results suggest that plastic bottles (Coke and Sams) were similar to the industry standard container (D27) as well as the polybag. However, root spiraling associated with polybags suggests that they are less desirable for producing quality seedlings. The superior performance of plastic bottles to polybags and their low-cost and availability relative to other modern alternatives makes them a suitable container type to produce quality seedlings, particularly in developing regions.

### Bottle modifications can improve plant quality

In the *Bottle Modification* experiment, both spiral prevention and opacity resulted in significant morphological responses for Afghan pine (Tables 3 and 4, respectively). By the final nursery sampling period, we found that the control treatment (containers with no spiral prevention method), caused increases in root spiraling. This concurs with previous findings that seedlings grown in solid-wall containers with no means of root spiraling prevention produced a greater number of spiraled and deformed roots [41–42]. Regardless of opacity, modifying the plastic bottle using a side-slit resulted in gains in RCD, shoot volume, and shoot biomass compared to control bottles. This is in contrast to the findings of Ortega et al. [42] who reported lower shoot dry mass due to air-pruning in side-slit containers compared to solid-wall containers. The observed increase in shoot response to side-slits may be the result of better root medium conditions that promoted better gas exchange, as indicated by Al-zalzaleh [43], who similarly found improved shoot responses in air-slit containers compared to solid-wall containers for *Acacia saligna* and *Eucalyptus viminalis*. Donahue et al. [44] also reported that seedling growth was improved with better water movement, and good aeration. Restricted aeration in container medium reduces photosynthesis, translocation, and growth [45].

Bottle opacity also caused important changes in seedling morphology. Seedlings grown in black containers had a greater number of FOLRs and higher fibrosity compared to green containers (Table 4). This suggests that the higher light absorption of the black containers increased substrate temperatures and thus promoted root growth [46]. Media temperature records showed that black containers had greater daily maximum temperatures compared to the other two colors (data not shown). Black containers promoted root development, and clear containers resulted in a increase in shoot volume and shoot biomass compared to the black containers (Table 4). Similarly, Markham et al. [47] found that red maple seedlings produced taller shoots when grown in clear containers compared to black and green containers. In contrast, Blanchard and Runkle [48] reported that container opacity did not impact biomass development of two orchid cultivars, *Doritaenopsis* (White Moon) and *Phalaenopsis* (Sharon Bay). Higher media temperatures observed in the black (vs. clear) containers in our study may have promoted greater root development over shoot development.

Our results for Afghan pine shoot height concurred with Irmak et al. [49] who reported longer shoots in clear containers compared containers to black containers; they also found that substrate temperatures for clear containers were always optimal and more favorable for root growth compared to black containers, which exceeded 40°C. Black containers also had less algae on the inner wall compared to both clear and green containers (Fig 3). Similar to our findings, Blanchard and Runkle [48]. observed less algae growth on the interior surfaces of containers with higher opacity. For both of our experiments, plant performance was not affected by algal growth, but this effect may impact the longevity and durability of the plastic bottle containers, thus rendering them unusable for additional seedling crops.

After outplanting, the root spiraling prevention treatment had no effects on Afghan pine height growth, which conflicts with reports of shorter shoot height in side-slit containers compared to solid-wall containers [42]. However, in agreement with our findings, Rune [50] reported similar aboveground responses for *Pinus* sylvestris L. seedlings grown in solid-wall and side-slit containers six years after outplanting. Container opacity effects were significant for relative height growth (Fig 4); green containers produced larger root collar diameters compared to black, though relative growth analyses showed no treatment effects (Fig 4). In both of our experiments, field irrigation may have reduced the potential to detect differences in early outplanting performance, or the one year observation after outplanting was simply too short.

### Applications to reforestation

The collection and re-use of bottles commonly used for beverage distribution can provide a cheap and effective container for tree seedling growth, particularly for developing regions with limited access to expensive modern containers. The bottles produced quality deciduous (Arizona walnut) and conifer (Afghan pine) seedlings under controlled growing conditions; however, seedling producers should also consider the following if they plan to utilize bottles as seedling containers.

Our experiments were initiated in a climate-controlled greenhouse with access to clean water, quality growing media, and fertilizer. Some nurseries in developing countries may not have access to facilities that provide such ideal conditions for seedling growth and development, and this may yield differing results or require modifications to bottle preparation procedures we tested. Seedling producers should consider how bottles could be incorporated into their existing facilities, available resources, and seedling handling and storage methods, and it is recommended that a systematic method be used to help ensure similar results.

Black bottles did present a tradeoff of lower shoot volumes and biomass for a high number of FOLRs and root fibrosity, which may be desirable for arid regions that experience extended dry periods and low seedling survival. However, it should be noted that the green and clear bottles resulted in no differences in any of the total tree measures compared to the other opacity treatments. Black bottles also exhibited very low levels of algal growth, which may be a concern in nurseries that intend to re-use the containers for subsequent crops. However, elevated growing media temperatures may be a problem in nurseries without the ability to adequately control growing space temperatures [49], and appreciably more effort is required to modify the color of the bottles themselves. Most plastic bottles produced worldwide are typically green or clear. Incorporating a black or opaque coating on bottles will add labor to the modification process, and fewer bottle modifications required for seedlings production will make this a more attractive alternative to the polybag system.

These are a few issues we have identified that may vary by individual circumstances. Future research should examine the performance of a variety of native species, alternative media types from locally available resources, watering regimes, and the potential to re-use bottles over multiple seedling rotations in these bottle container types. Re-use of plastic bottles are estimated to work for 2–5 rotations depending on plastic material, media, and environmental conditions of the nursery. For example, the Coke bottles used in this study were more durable because the plastic was thicker and more rigid when compared to the Sams bottles. In contrast to potential re-use of plastic bottles, polybags are typically used only once.

## Conclusions

We provide the first evidence to suggest that use of plastic bottles as an alternative container type in production nurseries offer a potentially cost-effective opportunity for incorporation into reforestation and restoration programs, especially in developing countries that lack access to modern container types. Our results showed that bottle containers with minor modifications produced seedlings comparable to modern container types and seedlings had better root architecture compared to polybags. Use of side-slits in these bottle containers is a simple and effective means to prevent root spiraling and improve seedling root system quality. Production of seedlings with quality root systems will enhance outplanting survival and performance, particularly on degraded sites. Container opacity did not have important impacts on seedling above- and below-ground morphology. In warm temperature nursery conditions, seedlings may benefit from lighter color containers because of lower sunlight absorptive capacity and maintenance of optimum substrate temperature. In addition to providing an effective means to produce high quality seedlings for reforestation, use of these bottles as nursery containers will reduce consumption of plastic in the agricultural sector and provide a commendable alternative for waste management.

Future directions in this research should examine potential modifications during the manufacturing process that would minimize the need for manual modifications (i.e., side slits for root spiral control) to transform the bottle into a cheap, accessible, and effective seedling container. These modifications would be minor and unnoticeable to the beverage consumer, thereby keeping the branding image of the manufacturer intact. These modifications would include: 1) modifying the bottles with internal plastic ridges to control root spiraling; 2) adopting bottles with thinner plastic in areas required to be cut or punctured for removal of the top and puncture holes for drainage in the bottom; and 3) testing different plastics types in comparison to the petroleum-based industry standard, polyethylene terephthalate (PET).

## Supporting information

S1 Dataset Data used in all analyses. All shoot and root plant morphology data for container comparison and bottle modification experiments; media temperature data for bottle modification experiment; and field performance data for container comparison and bottle modification experiments.
(XLSX)

## References

[1] FAO (2015) Global forest resources assessment 2015: how are the world's forests changing? Food and Agriculture Organization of the United Nations Rome, Italy; 2015 http://www.fao.org/3/a-i4793e.pdf

[2] Radoglou K, Raftoyannis Y. Effects of desiccation and freezing on vitality and field performance of broadleaved tree species. Ann Forest Sci. 2001; 58:59–68.

[3] Gregorio NO, Herbohn J, Harrison S. The potential role of nurseries in improving access to high quality planting stock and promote appropriate silvicultural systems to improve the productivity of smallholder tree farms in Leyte, Philippines In: ACIAR Smallholder Forestry Project-Improving Financial Returns to Smallholder Tree Farmers in the Philippines. The University of Queensland, pp. 163–172; 2005.

[4] Gregorio NO, Harrison S, Herbohn J. Enhancing tree seedling supply to smallholders in Leyte Province, Philippines: an evaluation of the production system of government nursery sector and support to smallholder tree farmers. Small-scale Forestry. 2008; 7:245–261.

[5] Harrington JT, Mexal JG, Wagner AM, Parsons T. The state and challenges of conservation nurseries in Afghanistan. In: Haase DL, Pinto JR, Riley LE, technical coordinators. National Proceedings: Forest and Conservation Nursery Associations-2011. Fort Collins (CO): USDA Forest Service, Rocky Mountain Research Station. 2012; Proceedings RMRS-P-68:59–64.

[6] Jacobs DF, Oliet JA, Aronson J, Bolte A, Bullock JM, Donoso PJ, et al Restoring forests: What constitutes success in the twenty-first century? New Forest. 2015; 46:601–614.

[7] Liu Y, Chen Y, Zhang Z, Li X. Effects of fertilizer treatments on seedling growth and cold resistance of triploid *Populus tomentosa*. Journal of Beijing Forestry University. 2000; 22:38–44.

[8] Gao ZG, Liu JL, Zheng GX, Zhong ZL. Study on effect of PP333 on *Pinus massoniana* seedling quality. Journal of Zhejiang Forestry Science and Technology. 2007; 27:54.

[9] Li GL, Zhu Y, Li QM, Liu Y, Zou SQ, Huang YL. Effect of seedling age on the seedling quality and field performance of *Pinus koraiensis*. Scientia Silvae Sinicae. 2012; 48:35–41

[10] Takoutsing B, Tchoundjeu Z, Degrande A, Asaah E, Gyau A, Nkeumoe F, et al Assessing the quality of seedlings in small-scale nurseries in the highlands of Cameroon: the use of growth characteristics and quality thresholds as indicators. Small-scale Forestry. 2014; 13:65–77.

[11] Landis TD. The Target Plant Concept In: Dumroese RK, Luna T, Landis TD, editors. Nursery manual for native plants: A guide for tribal nurseries—Volume 1: Nursery management. Agriculture Handbook 730 Washington, D.C.: U.S. Department of Agriculture, Forest Service p. 15–31; 2009.

[12] Lapis A, Posadas J, Pablo N. Seedlings/planting materials: A nationwide supply and demand scenario. Canopy International 3–10; 2001.

[13] Jaenicke H. Good Tree Nursery Practices—Practical Guidelines for Research Nurseries. International Centre for Research in Agroforestry, Nairobi, Kenya, 95p; 1999.

[14] Mexal JG. Forest nursery activities in Mexico. In: Landis TD, South DB (technical coordination). National Proceedings: Forest and Conservation Nursery Associations-1996. Gen. Tech. Rep. PNW-GTR-389. Portland, OR. U.S. Department of Agriculture, Forest Service, Pacific Northwest Research Station. 1997; 228–232.

[15] Bell TIW. Effect of seedling container restrictions on the development of Pinus caribaea roots. In: Van Eerden E, Kinghorn J, editors. Proceedings of the root form of planted trees symposium. British Columbia Ministry of Forests, Canada, Forest Service Joint Report. 1978; 8: 91–95.

[16] Mexal JG, Phillips R, Cuevas-Rangel RA. Forest nursery production in United States and Mexico. Proceedings of International Plant Propagators Society. 1994; 44:327–331.

[17] Landis TD. Containers: types and functions In: Landis TD, Tinus RW, McDonald SE, Barnett JP. The Container Tree Nursery Manual, Volume 2 Agric. Handbk. 674 Washington, DC: U.S. Department of Agriculture, Forest Service: 1–39; 1990.

[18] Budy JD, Miller EL. Survival, growth, and root form of containerized Jeffery pines ten year after outplanting. In: Murphy PM, compiler. The challenge of producing native plants for the intermountain area: Proceedings: Intermountain Nurseryman's Association 1983 conference; 1983 August 8–11; Las Vegas, NV. General Technical Report INT-168. Ogden, UT: U.S. Department of Agriculture, Forest Service, Intermountain Forest and Range Experiment Station; 1984.

[19] Burdett AN, Coates H, Eremko R. Martin PAF. Toppling in British Columbia's lodgepole pine plantations: significance, cause and prevention. Forest Chron. 1986; 62:433–439. doi: 10.1210/jcem-62-2-4333941163

[20] Lindström A. Stability in young stands of containerized pine (P. sylvestris). Swedish University of Agriculture Sciences. Translation from Internet Report; 1990.

[21] Halter MR, Chanway CP, Harper GJ. Growth reduction and root deformation of containerized lodgepole pine saplings 11 years after planting. Forest Ecol Manag. 1993; 56:131–146.

[22] Cedamon ED, Mangaoang EO, Gregorio NO, Pasa AE, Herbohn JL. Nursery management in relation to root deformation, sowing and shading. In: Harrison SR, Herbohn JL, Suh J, Mangaoang E, Vanclay J, editors. Proceedings from the ASEM/2000/088 end of project workshop. The University of Queensland, Brisbane; 2004.

[23] Muriuki J, Muia B, Munyi A. New methods improve quality of tree seedlings. APA News Asia-Pacific Agroforestry Newsletter. 2007; 31:3–5

[24] Harrison S, Gregorio N, Herbohn J. A critical overview of forestry seedling production policies and practices in relation to smallholder forestry in developing countries. Small-scale Forestry. 2008; 7:207–223.

[25] Cedamon ED, Mangaoang EO, Gregorio NO, Pasa AE, Herbohn JL. Nursery management in relation to root deformation, sowing and shading. In: ACIAR Smallholder Forestry Project-Redevelopment of a Timber Industry Following Extensive Land Clearing: Proceedings from the End-of-Project Workshop, The University of Queensland; 2005.

[26] Stein WI. Naturally developed seedling roots of five western conifers. In: Van Eerden E, Kinghorn J, editors. Proceedings of the root form of planted trees symposium. British Columbia Ministry of Forests, Canada, Forest Service Joint Report. 1978; 8:28–35.

[27] Sanghi S. Use of plastic bags: factors affecting ecologically oriented behavior in consumers. Abhigyan. 2008; 26:3 available at: http://www.highbeam.com/doc/1G1-192438179.html. Accessed March 2013.

[28] Adane L, Muleta D. Survey on the usage of plastic bags, their disposal and adverse effects on environment: a case study in Jimma City, Southwestern Ethiopia. J Toxicol Environ Health Sci. 2011; 3:234–248.

[29] Bewket W. Biofuel consumption, household level tree planting and its implications for environmental management in the northwestern highlands of Ethiopia. East Afr Soc Sci Res Rev. 2005; 21:19–38.

[30] Gleick PH. Bottled and sold: The story behind our obsession with bottled water. Washington, DC: Island Press; 2010.

[31] Bilderback TE, Owen JS Jr., Warren SL. A Gravimetric Approach to Real-Time Monitoring of Substrate Wetness in Container-Grown Nursery Crops. In International Symposium on Growing Media. 2007; 819:317–324.

[32] Dumroese RK, Montville ME, Pinto JR. Using container weights to determine irrigation needs: a simple method. Native Plants Journal. 2015; 16:67–71.

[33] Soil Survey Staff, Natural Resources Conservation Service, United States Department of Agriculture. Web Soil Survey; 2016. Available online at http://websoilsurvey.nrcs.usda.gov/. Accessed December 14, 2016.

[34] Tanaka Y, Walstad JD, Borrecco JE. The effect of wrenching on morphology and field performance of Douglas fir and loblolly pine seedlings. Can J Forest Res. 1976; 6:453–458.

[35] Thompson BE. Seedling morphological evaluation: what you can tell by looking In: Duryea ML, editor. Evaluating seedling quality: Principles, procedures, and predictive ability of major tests. Oregon State University, Corvallis, OR 1985; 59–71.

[36] Gasvoda DS, Tinus RW, Burr KE, Trent A. Monitoring the temperature of tree seedlings with the thermochron iButton Data Logger. USFS Technology and Development Program; 2002 Available in: http://fsweb.mtdc.wo.fs.fed.us/cgi-bin/enter.pl?link=pubs/htmlpubs/htm02242311/

[37] Dumroese RK, Wenny DL. An assessment of ponderosa pine seedlings grown in copper-coated polybags. 1997; Tree Planters' Notes 48:60–64.

[38] Aldrete A, Mexal JG, Phillips R, Vallotton AD. Copper coated polybags improve seedling morphology for two nursery-grown Mexican pine species. Forest Ecol Manag. 2002; 163:197–204.

[39] Davis RE, Whitcomb CE. Effects of propagation container size on development of high quality tree seedlings. Proceedings of International Plant Propagators Society. 1975; 25:448–453.

[40] Grossnickle SC. Importance of root growth in overcoming planting stress. New Forest. 2005; 30:273–294.

[41] Marshall MD, Gilman EF. Effects of Nursery Container Type on Root Growth and Landscape Establishment of *Acer rubrum* L. Journal of Environmental Horticulture. 1998; 16:55–59.

[42] Ortega U, Majada J, Mena-Petite A, Sanchez-Zabala J, Rodriguez-Iturrizar N, Txarterina K, et al Field performance of *Pinus radiata* D. Don produced in nursery with different types of containers. New Forest. 2006; 31:97–112.

[43] Al-Zalzaleh H. The effect of container type and soil substrates on growth and establishment of selected landscape trees. Scientific Papers-Series B, Horticulture. 2013; 57:255–260.

[44] Donahue RL, Miller RW, Shickluna JC. An introduction to soils and plant growth. Englewood Cliffs, Prentice Hall, New Jersey; 1983.

[45] Sutherland RJ, Day RJ. Container volume affects survival and growth of White Spruce, Black Spruce and Jack Pine Seedlings. North J Appl For. 1988; 5:185–189.

[46] Ingram DL. Characterization of temperature fluctuations and woody plant growth in white poly bags and conventional black containers. HortScience. 1981; 16:762–763.

[47] Markham JW, Bremer DJ, Boyer CR, Schroeder KR. Effect of container color on substrate temperatures and growth of red maple and redbud. HortScience. 2011; 46:721–726.

[48] Blanchard MG, Runkle ES. Container opacity and media components influence rooting of potted *Phalaenopsis* and *Doritaenopsis* orchids. In International Workshop on Ornamental Plants. 2007; 788:115–120.

[49] Irmak S, Haman DZ, Irmak A, Jones JW, Tonkinson B, Burch D, et al Root zone temperatures of *Viburnum odoratissimum* grown in the multipot box system and conventional systems: Measurement and analyses of temperature profiles and predicting root zone temperatures. HortScience. 2005; 40:808–818.

[50] Rune G. Slits in container wall improve root structure and stem straightness of outplanted Scots pine seedlings. Silva Fenn. 2003; 37:333–342.

